# Indian Hemp as an Oxytoxic

**Published:** 1851-03

**Authors:** 

**Affiliations:** Edinburgh


*Indian Hemp as an Oxytoxic.* By Dr. Simpson, Edinburgh.—Dr. Simpson stated, that, in the early part of the winter session, he had given Indian hemp (Cannabis Indica) in several cases of tedious labor, with the view of ascertaining if it possessed any oxytoxic effect (like ergot of rye) in increasing and exciting the parturient action of the uterus. He had been induced to try the effects, if any, of Indian hemp during labor, in consequence of Dr. Churchill stating, that it possessed powers similar to those of ergot of rye in arresting hemorrhage, when dependent upon congested states of the *unimpregnated* uterus. In the few cases of labor in which it was tried, parturient action seemed to be very remarkably and distinctly increased after the exhibition of the hemp; but far more extensive and careful experiments would be required, before a definite opinion could be arrived at relative to its possession of oxytoxic powers, and their amount.—*Monthly Journal.*

